# Potentiation of NMDA receptor-mediated transmission in striatal cholinergic interneurons

**DOI:** 10.3389/fncel.2015.00116

**Published:** 2015-04-09

**Authors:** Manfred J. Oswald, Jan M. Schulz, Wolfgang Kelsch, Dorothy E. Oorschot, John N. J. Reynolds

**Affiliations:** ^1^Department of Anatomy and the Brain Health Research Centre, University of OtagoDunedin, New Zealand; ^2^Central Institute of Mental Health, Medical Faculty Mannheim, Heidelberg UniversityMannheim, Germany

**Keywords:** cholinergic interneuron, plasticity, NMDA, dopamine, reward, pause response

## Abstract

Pauses in the tonic firing of striatal cholinergic interneurons (CINs) emerge during reward-related learning in response to conditioning of a neutral cue. We have previously reported that augmenting the postsynaptic response to cortical afferents in CINs is coupled to the emergence of a cell-intrinsic afterhyperpolarization (AHP) underlying pauses in tonic activity. Here we investigated in a bihemispheric rat-brain slice preparation the mechanisms of synaptic plasticity of excitatory afferents to CINs and the association with changes in the AHP. We found that high frequency stimulation (HFS) of commissural corticostriatal afferents from the contralateral hemisphere induced a robust long-term depression (LTD) of postsynaptic potentials (PSP) in CINs. Depression of the PSP of smaller magnitude and duration was observed in response to HFS of the ipsilateral white matter or cerebral cortex. In Mg^2+^-free solution HFS induced NMDA receptor-dependent potentiation of the PSP, evident in both the maximal slope and amplitude of the PSP. The increase in maximal slope corroborates previous findings, and was blocked by antagonism of either D1-like dopamine receptors with SCH23390 or D2-like dopamine receptors with sulpiride during HFS in Mg^2+^-free solution. Potentiation of the slower PSP amplitude component was due to augmentation of the NMDA receptor-mediated potential as this was completely reversed on subsequent application of the NMDA receptor antagonist AP5. HFS similarly potentiated NMDA receptor currents isolated by blockade of AMPA/kainate receptors with CNQX. The plasticity-induced increase in the slow PSP component was directly associated with an increase in the subsequent AHP. Thus plasticity of cortical afferent synapses is ideally suited to influence the cue-induced firing dynamics of CINs, particularly through potentiation of NMDA receptor-mediated synaptic transmission.

## Introduction

Modulation of acetylcholine and dopamine signaling is important for associative and goal-driven motor learning (Aosaki et al., 1994a; Morris et al., 2004; Joshua et al., 2008; Apicella et al., 2011). The effect of these neurotransmitters on learning is mediated in part through the control of striatal synaptic plasticity (Calabresi et al., 2007; Kreitzer and Malenka, 2008; Shen et al., 2008). Corticostriatal synapses onto projection neurons (SPNs) represent a major substrate for reward-related learning (Reynolds et al., 2001; Yin et al., 2009). Synaptic plasticity has also been described in striatal cholinergic interneurons (CINs; Suzuki et al., 2001; Reynolds et al., 2004; Fino et al., 2008).

CINs are thought to represent the population of striatal tonically active neurons (TANs) that briefly pause firing in response to primary rewards or aversive stimuli and acquire pause responses to conditioned cues (Aosaki et al., 1994b; Apicella et al., 1997; Apicella, 2007; Morris et al., 2004; Joshua et al., 2008; reviewed in Goldberg and Reynolds, 2011; Schulz and Reynolds, 2013). The pause response coincides with bursts of dopaminergic neuron firing (Morris et al., 2004; Joshua et al., 2008) and requires intact dopamine signaling in the striatum for its continued expression (Aosaki et al., 1994a). Iontophoretic application of D2 dopamine receptor antagonists in behaving primates blocked the pause response to reward-predicting cues, but in about 65% of recorded TANs only (Watanabe and Kimura, 1998), suggesting that dopamine does not work alone in driving the pause response.

Much focus over the last 10 years has been on the cellular mechanisms underlying the pause response, and in particular the relative role of glutamatergic and dopaminergic mechanisms. Various reward-predicting cues that are known to result in dopamine release typically evoke a multiphasic response in primate TANs, with an initial excitation preceding the pause response in 38% of neurons (Doig et al., 2014). A similar proportion of CINs displayed a burst-pause response on direct phasic optogenetic activation of dopaminergic terminals in the ventral mouse striatum, however CIN firing pauses preceded by an initial excitation were blocked by glutamate receptor antagonists (Wieland et al., 2014). Optogenetic activation of dopaminergic terminals in the shell region of the nucleus accumbens similarly evoked burst-pause responses in CINs that were blocked by AMPA and NMDA receptor antagonists (Chuhma et al., 2014). One explanation for the persistence of burst-pauses following direct activation of dopamine terminals in the presence of dopamine antagonism is the co-release of glutamate. However, this mechanism is prominent only in the ventral striatum (Hnasko et al., 2010; Stuber et al., 2010); phasic optogenetic stimulation of dopaminergic terminals in the dorsal striatum accordingly did not evoke an initial burst response before the CIN firing pause (Chuhma et al., 2014). Thus, the mechanisms underlying phasic burst firing of CINs in the dorsal striatum remain to be elucidated.

CINs receive excitatory glutamatergic inputs from the cerebral cortex primarily at distal dendrites (Dimova et al., 1993; Thomas et al., 2000; Sizemore et al., 2010; Doig et al., 2014) and proximally as well as distally from intralaminar thalamic nuclei (Lapper and Bolam, 1992). Cortical afferents arise from two distinct projection pathways (Wilson, 1995; Reiner et al., 2010). One is an ipsilateral collateral input from layer 5 neurons projecting to the brainstem and spinal cord via the pyramidal tract (PT-type) (Cowan and Wilson, 1994; Wright et al., 2001; Reiner et al., 2003). The other originates from intratelencephalic (IT-type) corticostriatal neurons that cross the midline and target striata in both hemispheres (Wilson, 1987; Wright et al., 2001; Reiner et al., 2003).

Multiphasic firing responses in CINs *in vivo* are evoked on stimulation of excitatory inputs from the cerebral cortex or thalamus (Reynolds et al., 2004; Schulz et al., 2011; Schulz and Reynolds, 2013; Doig et al., 2014). We have previously shown that excitatory inputs from the cortex evoke a cell-intrinsic afterhyperpolarization (AHP; Reynolds et al., 2004; Oswald et al., 2009). In addition, we demonstrated that visual activation of the subcortical tecto-thalamo-striatal pathway *in vivo* pauses CIN firing, likely through an intrinsic AHP (Schulz et al., 2011). Hence we hypothesized that plasticity of afferent synapses onto CINs will modulate the magnitude of the subsequent AHP. We show that cortical afferent synapses onto CINs exhibit bidirectional plasticity and that NMDA receptor potentials can be enhanced independently of AMPA/kainate receptor potentials with a Hebbian plasticity protocol. The enhanced NMDA receptor-mediated transmission gave rise to a prolonged depolarization that reliably triggered an AHP and is well suited to influence phasic firing properties of CINs.

## Materials and Methods

### Ethical Approval

All procedures involving live animals were approved by both local Animal Ethics Committees and were in accordance with the New Zealand Animal Welfare Act 1999.

### Slice Preparation

Acute brain slices were prepared from 43 P14–P24 male Wistar rats following decapitation under deep pentobarbital anesthesia (100 mg/kg intraperitoneal). Brains were perfused transcardially with ice-cold dissection solution (in mM: 225 sucrose, 10 glucose, 2.5 KCl, 7 MgCl_2_, 0.5 CaCl_2_, 28 NaHCO_3_, 1 NaH_2_PO_4_; bubbled with 95% O_2_ and 5% CO_2_). Slices (300–400 μm) were cut using a vibratome (VT1000S, Leica, Nussloch, Germany) at an oblique angle of ≈ 30° to the horizontal plane, in order to maximally preserve corticostriatal connections in both hemispheres (Oswald et al., 2009). Slices were transferred to artificial cerebrospinal fluid (ACSF) consisting of (mM): 125 NaCl, 2 MgSO_4_, 2 CaCl_2_, 2.5 KCl, 10 glucose, 26 NaHCO_3_, 1 NaH_2_PO_4_; bubbled with 95% O_2_ and 5% CO_2_. After cutting, slices were kept for the initial 45 min at 35°C, and then at room temperature for a minimum recovery period of 1 h.

### Electrophysiological Recording

For recording, slices were transferred to a temperature-controlled recording chamber (TC-2, Bioscience Tools, San Diego, CA) perfused with oxygenated ACSF (2 mL/min, 33°C). Striatal cholinergic interneurons were visualized using infrared-differential interference contrast optics (BX51WI, Olympus Optical, Tokyo, Japan) and an IR-1000 CCD camera (DAGE-MTI, Michigan City, IN, USA). Recording pipettes were prepared from borosilicate glass capillaries (1.5 mm outer diameter, 0.86 mm inner diameter; Harvard Apparatus, Edenbridge, UK) using a horizontal pipette puller (P87; Sutter Instruments, Novato, CA, USA). Pipettes had a resistance of 5–7 MΩ when filled with a solution containing (mM): 132 K-gluconate, 6 KCl, 6 NaCl, 2 Na_2_ATP, 0.4 Na_2_GTP, 2 MgCl_2_ and 10 HEPES (pH 7.4, 290–300 mOsm/L). Current-clamp recordings were made in the whole-cell configuration using a MultiClamp 700B amplifier (Molecular Devices, Union City, CA, USA), with series resistance compensation of 15–22 MΩ. The signals were lowpass filtered at 4 kHz and digitized at 20 kHz (1322A Digidata and pClamp9 acquisition software; Molecular Devices).

### Electrical Stimulation and Drug Application

Postsynaptic potentials (PSP) were evoked by electrical stimulation via a bipolar stereotrode (~0.2 MΩ; MicroProbe, Gaithersburg, MD) within the cerebral cortex or white matter in the hemisphere contralateral or ipsilateral to the recording site in the dorsolateral striatum, as previously described (Oswald et al., 2009). Biphasic stimuli (0.1 ms duration, up to 1 mA) controlled by a Master-8 pulse generator (A.M.P.I, Jerusalem, Israel) and a stimulus isolator (A13–75, Coulbourn Instruments, Allentown, PA, USA) were applied every 5 s for a baseline period of 10–15 min. CINs were recorded in current-clamp mode at I_*m*_ = 0 pA. An intracellular current pulse (100 pA, 10 ms) was delivered 1.75 s following each test stimulus to monitor input resistance throughout the experiment. To induce synaptic plasticity, a high frequency stimulus (HFS, 0.5 ms biphasic pulses delivered at 100 Hz over a 500 ms period and repeated every 10 s for six times) was paired with a 600 ms current pulse (100–350 pA) to ensure that CINs were active (8–20 Hz spike rate) during the period of synaptic input. Drugs were obtained from Tocris Bioscience (Bristol, UK), dissolved in ACSF on the day of the experiment, and bath applied for a 5–8 min period preceding and during the HFS. For a subset of experiments the cerebral cortex was removed by dissection just superior to the corpus callosum after transfer of the slice to the recording chamber.

Voltage-clamp recordings were performed with a HEKA EPC-10USB amplifier and acquired with Patchmaster software (HEKA). For these experiments the ACSF contained (in mM): 125 NaCl, 2 CaCl_2_, 2.5 KCl, 15 glucose, 25 NaHCO_3_, 1.25 NaH_2_PO_4_, and pipettes were filled with a solution containing (mM): 120 K-gluconate, 15 KCl, 7 Na_2_-phosphocreatine, 4 MgATP, 0.3 Na_2_GTP, 4 MgCl_2_ and 10 HEPES (pH 7.2, 290–300 mOsm/L). Pipette resistances ranged from 3–5 MΩ. CNQX (Biotrend, Germany) was dissolved at 1,000X in DMSO and added to the bath solution 10–12 min before the HFS. The membrane potential was clamped to −65 mV and biphasic stimuli (0.1–0.2 ms, 100–300 μA) applied to the ipsilateral corpus callosum through a low resistance glass pipette filled with ACSF. Input resistance was monitored by applying a voltage step (−10 mV, 250 ms) 1.5 s before the electrical stimulation. The cell was switched to current-clamp for plasticity induction, pairing HFS with intracellular current injection as described above.

### Data Analysis

Data were analyzed offline using MATLAB 7.8 with Signal Processing 6.11 and Statistics 7.1 Toolboxes (The MathWorks, Natick, MA, USA). Cell input resistance (R*_i_*) was determined from the regression slope of the peak membrane potential in response to 500 ms long step current pulses (−60 to +20 pA) at the start of each experiment, and was assessed also throughout the experiment by monitoring the peak amplitude responses to brief current pulses. Cells were discarded if R*_i_* changed by more than 20%. The strength of synaptic transmission was assessed from the maximal PSP amplitude and the slope, determined as the maximal value of a linear fit to the ascending phase of the PSP using a 1 ms sliding window (Schulz et al., 2010). The time difference between stimulation and the center of the 1 ms-long linear fit of the maximum slope was measured as the slope latency. The time from maximum slope to PSP peak amplitude was used as the time to peak measurement. The Lilliefors test was used to test if data followed a normal distribution for statistical analysis. The non-parametric paired signed rank test was used to analyse time to peak data which was the only data set not normally distributed. The probability level for statistical significance was set at *P* = 0.05. The plasticity data of each neuron was averaged over 1 min periods and normalized to the average of a 5 min control period preceding the HFS. A paired *t*-test was used to test for statistical significance in a mean change from baseline during the 20 min period following the HFS. Single episode data of corresponding 5 min control and 16–20 min post-HFS periods were used for the mean variance analysis. The input resistance over the same time period did not change significantly in any of the groups, and there was no correlation between input resistance and either PSP slope or amplitude when assessed with a linear regression analysis. Values are expressed as mean ± standard error (SEM) unless stated otherwise.

PSP and AHP areas were measured in individual episodes as previously described (Oswald et al., 2009). Each trace was lowpass filtered (20 Hz; 3rd order Butterworth), down sampled to 1 kHz and high pass filtered (0.2 Hz; 3rd order Butterworth) to obtain detrended smoothed episodes. The mean reference potential was computed from three periods in each episode: (i) immediately before the stimulation; (ii) 1.5 s after the stimulation; and (iii) at the end of each 3 s episode following the intracellular current injection. Episodes were excluded from analysis if the baseline was unstable, as indicated by the SD of the reference potential >0.25 mV, or if an action potential was evoked. Areas under the PSP (mV*ms) were measured from the start of the stimulus to the time at which the signal returned to the reference potential. At that point, the AHP was considered to commence. AHP areas were computed in the same manner from the above defined time point to the time at which the signal crossed the reference level again. Both PSP and AHP potentials were calculated for a minimum of 100 ms to avoid an early termination of the process due to noise. The mean areas of the 20 min post-tetanus period were normalized to the mean of the preceding 5 min control period. Linear regression analysis was used to test the relationship between plasticity-induced changes in PSP and AHP magnitudes across all neurons from all groups. The same parameters were also expressed as group mean vectors to visualize treatment effects.

## Results

**Figure 1:**
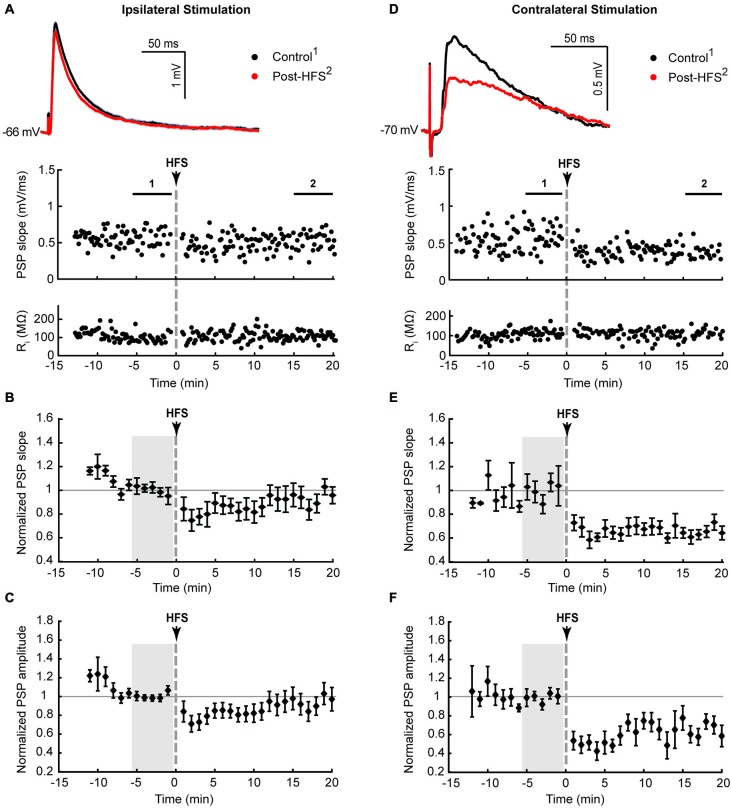
**Ipsilateral (A–C) and contralateral (D–F) cortical high frequency stimulation (HFS) depressed excitatory synaptic responses in striatal cholinergic interneurons. (A,D)** Single episode data of maximal postsynaptic potentials (PSP) slope and input resistance of a representative neuron, and average PSP waveforms of the time periods indicated. Group averages of maximal PSP slope **(B,E)** and amplitude **(C,F)** normalized to a 5 min stable control period (gray shaded area) before the HFS. A short lasting synaptic depression was apparent on ipsilateral stimulation (*n* = 12, *P* = 0.22 **(B)** and *P* = 0.14 **(C)**, paired *t*-test for the entire 20 min post-HFS period). The synaptic depression lasted over the entire 20 min post-HFS period upon selective stimulation of commissural cortical inputs in the contralateral hemisphere (*n* = 7, *P* < 0.001 **(E,F)**).

**Figure 2:**
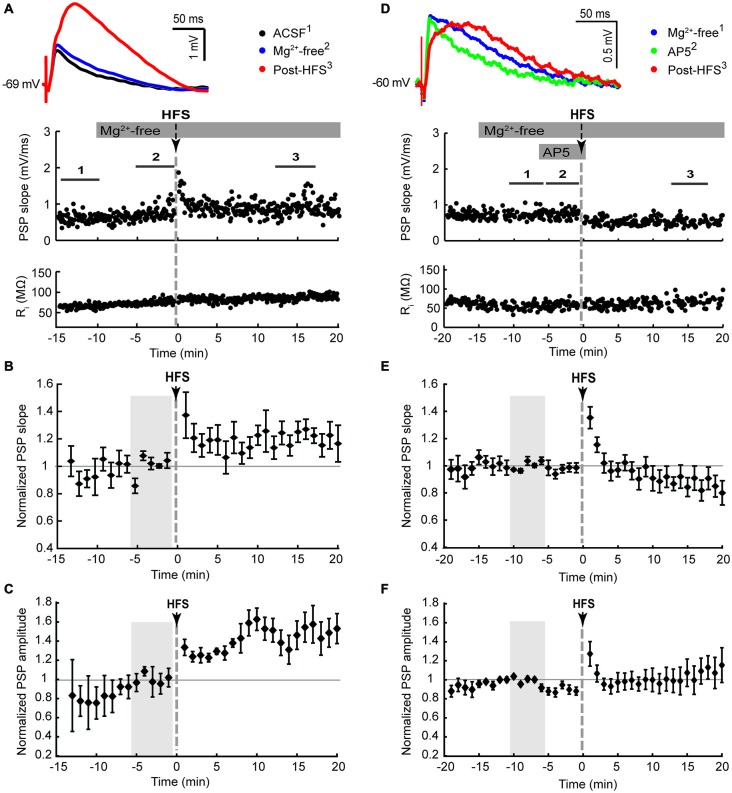
**NMDA receptor-dependent potentiation of cortico-striatal inputs to striatal cholinergic interneurons in Mg^2+^-free solution**. Ipsilateral cortical HFS in Mg^2+^-free ACSF potentiated PSPs **(A–C)**, which was blocked by the NMDA receptor antagonist AP5 (50 μM, **(D–F)**). **(A,D)** Single episode data of maximal PSP slope and input resistance of a representative neuron, and average PSP waveforms of the time periods indicated. Group averages of maximal PSP slope **(B,E)** and amplitude **(C,F)** normalized to a 5 min stable control period in Mg^2+^-free ACSF (gray shaded area) before either HFS alone or drug treatment followed by HFS. Potentiation of PSP slope and amplitude (*n* = 7, *P* < 0.05 **(B,C)**, paired *t-*test for the entire 20 min post-HFS period) were blocked in the presence of AP5 during the HFS (*n* = 8, *P* = 0.34 **(B)** and *P* = 0.79 **(C)**).

To selectively activate glutamatergic inputs to CINs we cut brain slices at an oblique angle between the coronal and horizontal planes with both hemispheres intact, and placed stimulating electrodes in the corpus callosum or the deep layers of the cerebral cortex. Brief electrical stimulation of fibers situated deep within the cerebral cortex and in the corpus callosum evoked depolarising PSPs in CINs of the dorsolateral striatum. PSP latencies remained constant upon varying stimulus intensities, as expected for a monosynaptic response; test response stimulus intensity was set to evoke PSP amplitudes of about half of the maximal response. We have shown previously that PSPs under these stimulation conditions in this preparation are mediated by both AMPA and NMDA type glutamate receptors and that inhibitory GABA_A_ receptor mediated potentials were not evoked (Oswald et al., 2009). PSPs evoked by ipsilateral stimulation (e.g., Figures 1A, 2A) had peak amplitudes ranging from 0.7 to 3.6 mV, with group averages ranging from 1.1 ± 0.4 to 1.5 ± 0.9 mV (mean ± SD, Table 1). Mean latencies from time of stimulation to PSP onset ranged from 5.2 ± 1.0 to 8.5 ± 2.8 ms in the ipsilateral stimulation groups. PSP rise times in the ipsilateral ACSF group ranged from 1.2 to 18 ms (median = 5.6 ms, Table 1).

**Table 1:** **Electrophysiological properties of recorded neurons**.

		Contralateral stimulation	Ipsilateral stimulation				
	Time^$^	ACSF (*n* = 7)	ACSF (*n* = 12)	Mg^2+^-free (*n* = 7)	Mg^2+^-free AP5 (*n* = 8)	Mg^2+^-free SCH23390 (*n* = 10)	Mg^2+^-free Sulpiride (*n* = 11)
		A	B	C	D	E	F
Input resistance (MΩ)		180 ± 73^§^	185 ± 53	184 ± 60	175 ± 53	207 ± 37	240 ± 38
PSP Latency (ms)		12.7 ± 3.5	8.0 ± 3.5	8.5 ± 2.8	5.2 ± 0.9	5.2 ± 1.0	7.1 ± 2.6
Time to peak (ms)^†^	Pre	15 (12, 16)	5.6 (3.9, 12)	4.7 (2.9, 31)	17 (9.3, 24)	20 (13, 22)	20 (14, 32)
	Post	20 (16, 31)	5.6 (3.8, 14)	34 (18, 47)*	29 (21, 35)	40 (21, 71) **	40 (31, 52)***
PSP peak amplitude (mV)
ACSF	Pre	0.8 ± 0.2	1.5 ± 0.9	1.1 ± 0.4	1.2 ± 0.4	1.5 ± 0.6	1.2 ± 0.2
Mg^2+^-free	Pre			1.5 ± 0.8	1.3 ± 0.5	1.6 ± 0.7	1.4 ± 0.3
Mg^2+^-free and Drug treatment	Pre				1.2 ± 0.4	1.7 ± 0.8	1.9 ± 0.5
Normalized PSP peak amplitude*^f^*	Post	0.6 ± 0.2*^c–f^*	0.9 ± 0.3*^cef^*	1.4 ± 0.3*^abe^*	1.0 ± 0.3*^acef^*	1.4 ± 0.4*^abdf^*	1.8 ± 0.5*^abde^*

*^$^5 min reference period before (Pre) or 16–20 min after (Post) the HFS; 1–20 min Post HFS period for normalized PSP peak amplitude averages*.

*^§^Data values represent the mean ± standard deviation except for ‘Time to peak’ data, which is median (1st quartile, 3rd quartile)*.

^†^Time from maximum slope to PSP peak; significant post-HFS differences from pre-HFS data are indicated (*P < 0.05,**P < 0.005,***P < 0.001, paired signed rank test).

*^f^Group differences indicated by superscripts (one-way ANOVA and Tukey’s least significant difference test, *P* < 0.05)*.

Previous studies described the potentiation of depolarising and hyperpolarising PSPs in CINs in response to high frequency stimulation (HFS) of the corpus callosum in sagittal rat brain slices (Suzuki et al., 2001) and hyperpolarising PSPs in response to intrastriatal HFS in horizontal rat brain slices (Bonsi et al., 2004). In these studies it was necessary to block GABA_A_ receptors with bicuculline in order to isolate glutamate-evoked depolarising PSPs. During initial trial experiments with bicuculline added to bi-hemispheric slices we observed the occurrence of polysynaptic EPSPs with latencies ranging from 25 to 150 ms in several CINs recorded from different slice preparations (*n* = 8, not shown). Since hyperpolarising PSPs were not evoked with our stimulation conditions, and PSP magnitudes evoked by a stimulus train in recordings without a polysynaptic component remained unchanged after blockade of either GABA_A_ or GABA_B_ receptors with bicuculline or CGP55845, respectively (Oswald et al., 2009), we tested for plasticity-inducing effects on PSPs in CINs with inhibitory microcircuits intact. Ipsilateral HFS (100 Hz, 6 × 500 ms at 10 s interval, paired with intracellular current injection to ensure action potential spiking during HFS) resulted in depression of the maximal PSP slope and amplitude lasting less than 10 min after HFS. When averaged over the entire 20 min post-HFS period, the normalized data did not reach statistical significance, highlighting the short-lasting nature of the plasticity (Figures 1A–C, PSP slope = 90 ± 7.4%, PSP amplitude = 87 ± 8.4%, *n* = 12).

Ipsilateral stimulation activates both PT-type and IT-type corticostriatal afferents anterogradely, and possibly also thalamostriatal fibers in a retrograde manner. To selectively activate IT-type corticostriatal afferents we stimulated commissural afferents in the contralateral hemisphere. Stable PSPs in CINs could be evoked from the contralateral hemisphere typically only in one or two slices per preparation. In comparison to ipsilateral stimulation, PSPs evoked by stimulation of commissural afferents in the contralateral corpus callosum were somewhat smaller in amplitude (cf. Figures 1A,D), ranging from 0.6 to 1.1 mV, exhibited longer latencies (12.8 ± 3.5 ms) in accord with the increased stimulation distance, and were slower to peak (median = 14.7 ms, Table 1). In contrast to ipsilateral stimulation, HFS of commissural IT-type afferents in isolation resulted in a robust long-term depression (LTD) of PSP maximal slope (Figure 1E, 69 ± 3.9%) and amplitude (Figure 1F, 61 ± 6.4%, *n* = 7). There were no changes in CIN input resistance during the 20 min post-HFS period (Figures 1A,D, 101.7 ± 1.9% after contralateral stimulation, *P* > 0.05), hence HFS-induced depression of the PSPs was due to a reduction in synaptic strength.

LTP, but not LTD, of excitatory inputs to striatal SPNs requires NMDA receptor activation (Shen et al., 2008). The absence of LTD in CINs in response to ipsilateral stimulation may be due to recruiting both PT-type and IT-type afferent pathways leading to enhanced activation of NMDA receptors in comparison to isolated activation of IT-type afferents from the contralateral hemisphere. We therefore tested if augmenting NMDA receptor activation would potentiate synaptic responses, similar to what has been reported for SPNs (Kerr and Wickens, 2001). To remove the voltage-dependent Mg^2+^ block from NMDA receptors the bath solution was switched to Mg^2+^-free ACSF after a stable PSP was elicited by ipsilateral stimulation. This increased the PSP amplitude on average by 39 ± 14% (*n* = 7, Table 1, Figures 2A,C). Ipsilateral HFS in Mg^2+^-free solution potentiated the PSPs in CINs. The maximal PSP slope increased on average by 21 ± 8% during the 20 min post-HFS period relative to the 5 min reference period in Mg^2+^-free ACSF (Figure 2B), whereas the PSP amplitude increased by 42 ± 12% (Figure 2C). The latter was mainly due to the appearance of a slow PSP component (Figure 2A), also reflected in a significant delay in the time to PSP peak compared to the pre-HFS reference period (Table 1, *P* < 0.05, paired signed rank test, *n* = 7).

To test if the potentiation of PSPs in Mg^2+^-free ACSF was dependent on NMDA receptor activation we added the NMDA receptor antagonist AP5 to the bath solution for 5 min before and during the HFS. Potentiation of the PSP slope and amplitude was no longer observed following HFS in the presence of AP5 (Figures 2D,E), indicating a reliance on NMDA receptor activation.

**Figure 3:**
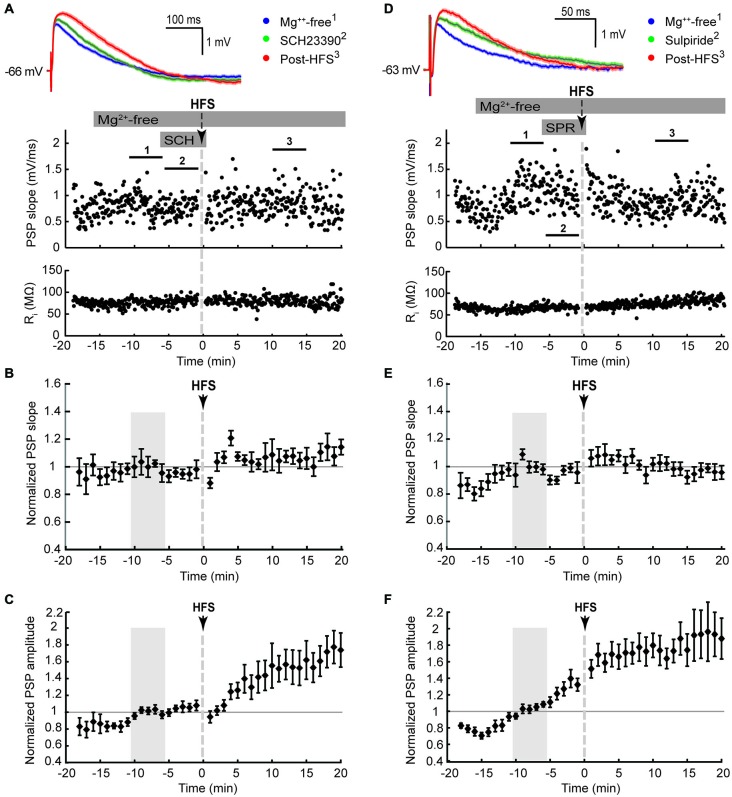
**D1-like (SCH23390, 10 μM, A–C) and D2-like (sulpiride, 10 μM, D,E) dopamine receptor antagonists blocked the potentiation of PSP maximal slope, but not amplitude, in striatal cholinergic interneurons recorded in Mg^2+^-free solution. (A,D)** Single episode data of maximal PSP slope and input resistance of a representative neuron, and average PSP waveforms of the time periods indicated. Group averages of maximal PSP slope **(B,E)** and amplitude **(C,F)** normalized to the 5 min control period in Mg^2+^-free ACSF (gray shaded area) before drug treatment and HFS. PSP maximal slopes did not potentiate in response to ipsilateral cortical HFS in Mg^2+^-free ACSF when D1-like **(B)**, *n* = 10, *P* = 0.12) or D2-like **(E)**, *n* = 11, *P* = 0.65) dopamine receptors were blocked, but PSP maximal amplitudes still potentiated **(C)**, *n* = 10, *P* < 0.05; **(F)**, *n* = 11, *P* < 0.001; paired *t*-tests for the entire 20 min post-HFS period).

We next tested if the activation of either D1-like or D2-like dopamine receptors was required for the Mg^2+^-free synaptic potentiation by adding their respective antagonists SCH23390 or sulpiride to the bath solution for 5 min before and during the HFS (Figure 3). Both treatments blocked the potentiation of the fast component represented by the maximal PSP slope (Figures 3B,E). However, the slow component, represented by the PSP amplitude, remained elevated over the course of the 20 min post-HFS period by 42 ± 14% with SCH23390 (Figure 3C, *P* < 0.01, *n* = 10) and by 75 ± 17% with sulpiride (Figure 3F, *P* < 0.001, *n* = 11). The time to the PSP peak was accordingly increased after HFS in the Mg^2+^-free SCH23390 and sulpiride groups (Table 1, *P* < 0.01 and *P* < 0.001, respectively, paired signed rank tests). The enhancing effect of D2 receptor blockade on the post-HFS PSP amplitude was likely contributed to by an increased PSP amplitude induced by sulpiride prior to HFS (Table 1, *P* < 0.01, one-way ANOVA and Tukey’s least significant difference test), potentially due to enhanced glutamate release at corticostriatal terminals (Bamford et al., 2004). However, the selective increase of the slow PSP amplitude component in response to D2 receptor blockade before the HFS may also indicate a direct modulation of the underlying conductance by D2 dopamine receptors.

**Figure 4:**
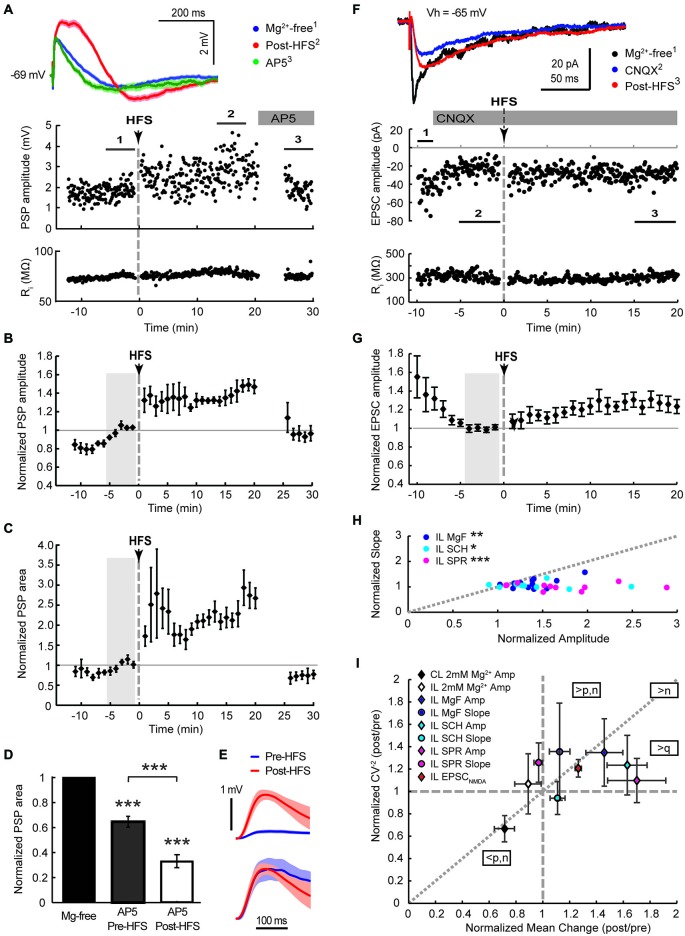
**Augmented NMDA receptor-mediated synaptic transmission dominates the HFS-induced potentiation of cortico-striatal inputs to cholinergic interneurons in Mg^2+^-free solution**. Ipsilateral stimulation in Mg^2+^-free solution in mono-hemispheric slices with trimmed cerebral cortex (**A–C**, current-clamp) or intact bi-hemispheric slices (**F–G**, voltage-clamp). **(A,F)** Single episode data of maximal amplitude and input resistance of a representative neuron, and average PSP waveforms of the time periods indicated. Group averages of maximal amplitude **(B,G)** and PSP area **(C)** normalized to the control period before HFS (gray shaded area). The increase in PSP amplitude and area (*n* = 5, *P* < 0.01 **(B,C)**, paired *t*-test for the entire 20 min post-HFS period) were neutralized after washing on the NMDA receptor antagonist AP5 (50 μM) from 20 min after the HFS (*n* = 4, *P* = 0.92 **(B)** and 0.11 **(C)**, paired *t*-test to Mg^2+^-free control condition). **(D)** Comparing the magnitude of PSP areas in the presence of AP5 normalized to that in Mg^2+^-free ACSF before HFS (gray, PSP area data from neurons in Figure 2F) and after HFS (open, data from Panel **(C)** and an additional neuron from the Mg^2+^-free ACSF group) revealed that NMDA receptor potentials contributed significantly more to the PSP following the HFS (35% reduction pre-HFS, *n* = 8; 77% reduction post-HFS, *n* = 5;***P < 0.001, paired and two-sample *t*-tests). **(E)** The average pre-HFS and post-HFS NMDA receptor potentials of all neurons in each group were revealed by subtracting average PSP waveforms in the presence of AP5 from that of the 5-min period before AP5 application (top, mean ± SEM) and normalized to the peak potential in order to compare the time course (bottom). **(F,G)** HFS potentiated EPSC_NMDA_ amplitudes isolated by blocking AMPA receptors with CNQX (10 μM, *n* = 11, *P* < 0.01 for the 20 min post-HFS period). **(H)** In Mg^2+^-free solution potentiation of the slow PSP amplitude component was pronounced in comparison to the PSP slope, indicated by points plotted below the gray-dotted line (normalized post-HFS data from cells in Figures 2A–C and with dopamine antagonists SCH23390 or sulpiride in Figure 3 (SCH and SPR, respectively);***P < 0.001,**P < 0.01, *P < 0.05, paired *t*-test). **(I)** Group summary of amplitude and PSP slope changes (16–20 min post-HFS normalized to the pre-HFS baseline period) plotted against the normalized squared inverse of the coefficient of variation (CV^−2^). According to quantal theory the parameters n (number of active release sites), p (release probability) and q (quantal size) determine postsynaptic response size. Individual parameter contributions to potentiated or depressed synapses can be estimated from the relationship between normalized CV^−2^ and the normalized mean change, as indicated (see Section Results). Amp, amplitude; CL, contralateral stimulation; IL, ipsilateral stimulation; MgF, Mg^2+^-free ACSF.

The slow dynamics of the increased PSP after HFS in Mg^2+^-free ACSF suggested the potentiation of NMDA receptor-mediated synaptic transmission, as reported for the hippocampal mossy fiber synapse on pyramidal neurons (Kwon and Castillo, 2008; Rebola et al., 2008). However, the slow PSP component could instead reflect enhanced recruitment of cortical afferents following HFS in response to orthodromic activation of cortical microcircuits. To test for this possibility, the ipsilateral cerebral cortex above the corpus callosum was dissected away from striatal brain slices. Single test stimuli were applied to the ipsilateral corpus callosum. HFS in Mg^2+^-free ACSF still potentiated the PSP amplitude recorded in CINs by 44 ± 7% (Figures 4A,B, *n* = 5), ruling out the possibility that enhanced di-synaptic recruitment of cortical afferents was underlying potentiation of the slow PSP component. Furthermore, the subsequent application of the NMDA receptor antagonist AP5 from 20 min post-HFS completely reversed the increase in PSP amplitude to baseline levels (Figures 4A,B), supporting the hypothesis that a selective increase of NMDA receptor-mediated synaptic transmission was underlying this effect.

A selective potentiation of NMDA receptor-mediated potentials was further supported by the examination of the AP5-sensitive proportion of the PSP area before and after the HFS. PSP areas were potentiated to 255 ± 29% of controls in Mg^2+^-free ACSF during the 20 min post-HFS period. Application of AP5 reduced the PSP area back to 73 ± 12% of controls (Figure 4C, *n* = 4). Overall, AP5 application 20–30 min after the HFS reduced the PSP area to 33 ± 5% of the potentiated PSP area during the preceding 5 min period (Figure 4D, *n* = 5). In contrast, AP5 application before the HFS reduced PSP areas recorded in Mg^2+^-free ACSF on average to 65 ± 4% of Mg^2+^-free controls (Figure 4D, *n* = 8). Hence, NMDA receptor potentials contributed significantly more to the PSP following the HFS (*P* < 0.001, two-sample *t*-test). In order to compare the NMDA receptor components of PSPs before and after the HFS, average PSP waveforms in the presence of AP5 were subtracted from those obtained during the 5-min period prior to AP5 application and averaged across all neurons in each group (Figure 4E, top). Normalizing the resulting waveforms by the peak amplitude revealed a similar time course of the NMDA receptor-mediated components before and after the HFS (Figure 4E, bottom). The fact that we observed similar NMDA receptor-mediated waveforms in CINs after blocking AMPA receptors with 10 μM CNQX (peak latency of 31 ms at an amplitude of 2.7 mV and 50 ms at an amplitude of 4.5 mV, not shown) lends support that we mainly monitored NMDA receptor mediated conductance changes by measuring the slow PSP peak component.

To test directly if NMDA receptors are potentiated in CINs we blocked AMPA/kainate channels with 10 μM CNQX in Mg^2+^-free solution and measured EPSC amplitudes in response to single test stimuli applied to the ipsilateral corpus callosum. After isolated EPSC_NMDA_ amplitudes reached a stable baseline, HFS was applied as before in current clamp and the cell immediately switched back to voltage clamp for continued monitoring of EPSC_NMDA_ amplitudes (Figure 4F). HFS on average increased EPSC_NMDA_ amplitudes to 123 ± 6% of the preceding baseline amplitude of −19, 9 ± 2.5 pA (Figure 4G, *P* < 0.01, *n* = 11). Subsequent addition of 50 μM AP5 reduced EPSCs to 4 ± 2% of the EPSC_NMDA_ amplitude (*n* = 4; data not shown) confirming that we were monitoring NMDA receptor conductances. Without blocking AMPA/kainate receptors, potentiation of the slow PSP component monitored in current clamp in Mg^2+^-free solution was greater than that of the fast PSP component (Figure 4H, *P* < 0.05, paired *t*-test, *n* = 7). This was even more pronounced when dopamine receptors were blocked during the HFS (Figure 4H).

In order to obtain some mechanistic insight, we conducted a mean variance analysis of the single episode data of the 15–20 min post-HFS and corresponding 5 min baseline periods. The distribution of postsynaptic amplitudes is determined by vesicular release probability (p), the number of release sites (n), and the quantal content (q). Changes in these parameters can be inferred by plotting the normalized squared inverse of the coefficient of variation against the normalized mean change (Sola et al., 2004; Figure 4I). Data points that distribute along the diagonal suggest that n changed mainly; q changed mainly if they fall on the horizontal axis without a change in the CV^−2^ (normalized CV^−2^ = 1) or in the sector between this axis and the diagonal; and a change in p is indicated if LTP points fall in the sector above the diagonal, and LTD points below the diagonal (Sola et al., 2004). Group averages of the potentiated slow PSP component and EPSC_NMDA_ fell in the sector that suggests an increase in q with a likely postsynaptic origin due to an increased availability of NMDA receptors. While potentiated fast and slow PSP components of the ipsilateral Mg^2+^-free stimulation group both displayed similar increases in normalized CV^−2^, this increase was greater than expected by an increase in release sites in the case of the fast PSP component, suggesting that an increase in presynaptic release probability was involved. The LTD group resulting from the contralateral HFS similarly appeared to involve a decrease in presynaptic release probability. Collectively these findings suggest that NMDA receptors were selectively up-regulated in the postsynaptic membrane of CINs in response to tetanic stimulation of cortical afferents in Mg^2+^-free conditions.

**Figure 5:**
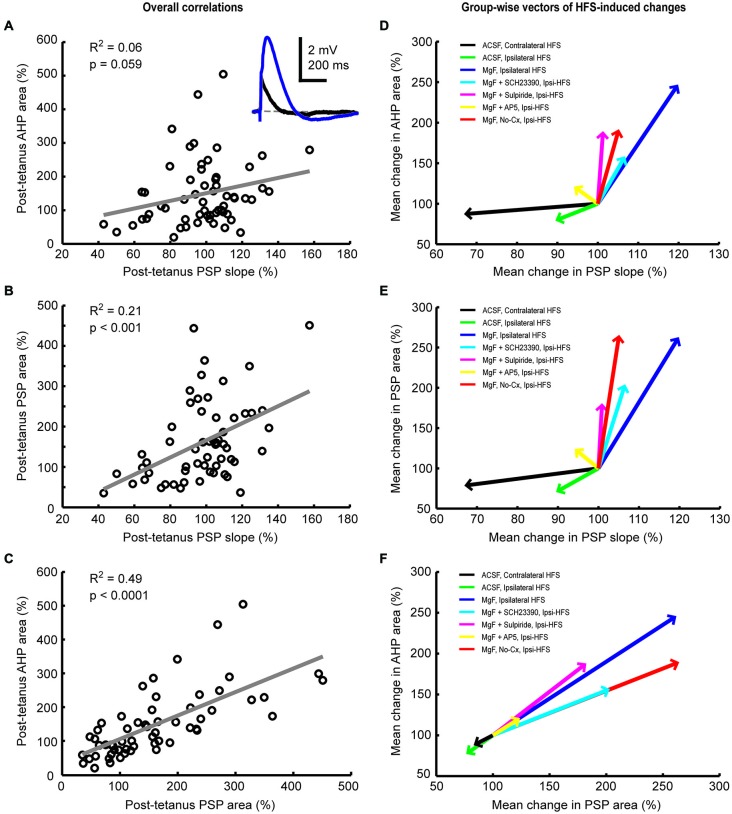
**Plasticity-induced changes in PSP and afterhyperpolarization (AHP) areas are correlated in striatal cholinergic interneurons**. Linear regression analysis of mean normalized PSP and AHP magnitudes for the entire 20-min period after the plasticity protocol across all groups **(A–C)** and vector plots of the grand mean for each experimental group **(D–F)**. A inset: Example average waveforms of a CIN in the Mg^2+^-free ACSF group before (blue) and after (red) HFS (resting membrane potential = −72 mV). PSP and AHP magnitudes were defined, respectively, as the area above and below the reference membrane potential indicated by the gray dashed line. The change in PSP slope was a poor predictor of the change in AHP area **(A,D)** and correlated reasonably well with the change in PSP area **(B,E)**. Changes in PSP and AHP areas were highly correlated **(C,F)**. This indicates that HFS-induced synaptic modulation involves NMDA receptors and was therefore not limited to AMPA/kainate receptor-mediated changes in postsynaptic conductances.

We have previously shown that activation of cortical (Reynolds et al., 2004) as well as thalamic (Schulz et al., 2011) excitatory afferents induces a depolarization-hyperpolarization sequence in CINs. The AHP is associated with a pause in tonic firing and is largely due to the cell-intrinsic activity of voltage-sensitive channels (Oswald et al., 2009; Schulz et al., 2011). Here, we measured the correlation between changes in PSP and AHP magnitudes to determine if augmentation of afferent synaptic inputs by plasticity mechanisms would lead to an enhancement of the intrinsic AHP. First, we found that plasticity-induced changes in PSP slopes were a relatively weak predictor of changes in AHP areas (Figures 5A,D, *R*^2^ = 0.06). Second, although HFS-induced changes in PSP slope were reasonably correlated with PSP area changes (Figures 5B,E, *R*^2^ = 0.21), plasticity-induced changes in PSP area itself were the best predictor of the change in AHP magnitude (Figures 5C,F, *R*^2^ = 0.49). Since the NMDA receptor-mediated PSP component greatly influenced the change in PSP area (Figure 4C) it also acts as a powerful modulator of the AHP.

## Discussion

The present study demonstrates a bi-directional plasticity at corticostriatal synapses onto CINs and shows that LTP induction is critically dependent on NMDA and dopamine receptor activation. Importantly we reveal for the first time that the NMDA receptor potential itself can be potentiated selectively, and independently of dopamine receptor activation, leading to a prolonged PSP that elicited an AHP of a magnitude proportional to the preceding depolarization (Figures 4, 5). We propose that potentiation of NMDA receptors in CINs will critically influence tonic as well as phasic firing activity.

### Synaptic Plasticity of the Fast PSP Component

PSPs evoked by cortical/thalamic stimulation in CINs *in vitro* are completely blocked by the ionotropic glutamate receptor antagonists (Oswald et al., 2009). Compared to AMPA receptors, NMDA receptors display slow rise-time and decay kinetics (Cull-Candy and Leszkiewicz, 2004; Granger et al., 2011) that significantly contribute to the amplitude and area of the PSP, but not to the maximal slope. The dissociative effect of dopamine receptor blockade on the potentiation of fast and slow PSP components in Mg^2+^-free solution, combined with the mean variance analysis, suggest that NMDA receptors are regulated independently of AMPA/kainate receptors as described for other cell types (Rebola et al., 2010).

We identified a requirement for D1- and D2-like dopamine receptors for LTP of the fast PSP component as measured by the maximal PSP slope. HFS in Mg^2+^-free solution stimulates dopamine release by activation of presynaptic NMDA receptors located on dopaminergic terminals (Tarazi and Baldessarini, 1999). CINs express the D1-like dopamine receptor (Yan and Surmeier, 1997; Berlanga et al., 2005) that has an excitatory influence on CINs (Aosaki et al., 1998; Centonze et al., 2003), and D2 receptors that inhibit the hyperpolarization-activated cation current (I*_H_*) (Deng et al., 2007). D1-like receptor activation has been identified by Suzuki et al. (2001) as a requirement for the potentiation of the PSP slope in CINs. Similarly, D2 receptor activation likely favors potentiation of synaptic transmission in CINs, as dendritic I*_H_* has been found to form an inhibitory constraint on the induction of LTP in hippocampal CA1 pyramidal neurons (Tsay et al., 2007).

NMDA receptor activation was essential for LTP induction in CINs. Using a spike-timing dependent protocol, Fino et al. (2008) also found a requirement for NMDA receptor activation in the induction of synaptic potentiation in CINs. In contrast, Suzuki et al. (2001) reported that NMDA receptor activation was not required, possibly due to the presence of Ca^2+^-permeable AMPA channels in CINs. The discrepancy in NMDA receptor dependency might be due to differing Mg^2+^ concentrations and the use of bicuculline in the study by Suzuki et al. (2001). In addition to blocking GABA_A_ receptors, bicuculline also blocks dendritic Ca^2+^-activated SK channels (Villalobos et al., 2004) that blunt EPSPs through a local shunting mechanism on calcium entry into dendritic spines (Ngo-Anh et al., 2005). Dendritic calcium entry in the Suzuki study may have been mediated mainly through Ca^2+^-permeable AMPA and voltage-dependent Ca^2+^ channels, as Mg^2+^ was present to oppose NMDA receptor conductance. Since our study was conducted in the absence of bicuculline, dendritic Ca^2+^-activated SK channels were able to blunt EPSPs effectively and the threshold for synaptic potentiation was not reached. Efficient LTP induction in our case was achieved by removing the Mg^2+^ block of NMDA receptors in order to boost dendritic calcium entry and hence was expected to depend on NMDA receptor activity. In addition, the increase in membrane depolarization due to the full availability of NMDA receptors in Mg^2+^-free solution would also allow for calcium influx through voltage-gated Ca^2+^ channels.

It is well established that HFS of corticostriatal afferents paired with cellular depolarization induces LTD in striatal spiny projection neurons (Wang et al., 2006; Calabresi et al., 2007; Kreitzer and Malenka, 2008). This LTD depends on the activation of D2 dopamine receptors and retrograde endocannabinoid signaling to suppress vesicular release probability on activation of CB1 receptors at presynaptic terminals. The LTD induced in CINs by the selective stimulation of IT-type afferents appears to be of a similar nature, since a presynaptic expression mechanism involving reduced release probability was indicated by the mean variance analysis. It appears that combined activation of PT-type and IT-type afferents blunted the generation of endocannabinoids postsynaptically. In addition to dopaminergic terminals, NMDA receptors are localized also on corticostriatal axon terminals (Tarazi and Baldessarini, 1999). Perhaps an enhanced presynaptic Ca^2+^ flux in Mg^2+^-free solution leads to a lasting increase in vesicular release probability as indicated by the mean variance analysis for the fast PSP component. This possibility is speculative at this stage and the exact mechanisms involved remain to be elucidated.

### Potentiation of the Slow PSP Component

Our experiments demonstrated that the slow NMDA receptor potential in CINs was potentiated independently of the fast AMPA/kainate receptor-mediated PSP component. Although LTP is typically associated with an increase in AMPA receptors within the postsynaptic density in other brain areas (Malenka and Bear, 2004), it has become apparent that NMDA receptors themselves can be trafficked in and out of the synapse in an activity-dependent manner (Lau and Zukin, 2007; Paoletti et al., 2013). For instance, protein kinase C-dependent insertion of NMDA receptors underlies NMDA receptor-dependent LTP of Schaeffer collateral-commissural inputs to CA1 pyramidal neurons (Grosshans et al., 2002). Hence the number of postsynaptic NMDA receptors is a principal component that is regulated in certain types of synapses.

In midbrain dopamine neurons, synapse-specific potentiation of NMDA receptor transmission was uncovered on pairing HFS of excitatory inputs with postsynaptic burst firing (Harnett et al., 2009). Similarly, robust and selective LTP of NMDA receptors at mossy fiber synapses onto CA3 pyramidal neurons can be induced with short bursts of stimulation (Kwon and Castillo, 2008; Rebola et al., 2008). LTP induction of NMDA receptor responses was expressed post-synaptically in these cases, and required group 1 mGluR and NMDA receptor activation in concert with a post-synaptic rise of intracellular calcium (Rebola et al., 2008). We found that NMDA receptor activation was essential for potentiating NMDA receptor mediated synaptic transmission in CINs. It is likely that we also induced a significant rise in post-synaptic calcium during LTP induction as we paired presynaptic HFS with postsynaptic depolarization to ensure action potential firing. However, action potential firing induced by somatic current injection during the HFS in standard ACSF appeared to be insufficient to remove the voltage-dependent Mg^2+^ block at distal dendrites. This may be due to the 2 mM Mg^2+^ used in our standard ACSF being double, or close to double the concentration used by other investigators (Fino et al., 2008; Kwon and Castillo, 2008; Rebola et al., 2008; Shen et al., 2008; Harnett et al., 2009). The Mg^2+^-block *in vivo* lies between these two extremes (Shu et al., 2003). Hence, repetition of highly convergent activity expected to occur during attentive states (Saalmann et al., 2012) may activate NMDA receptors sufficiently to increase cytoplasmic calcium in CIN dendrites, leading to the potentiation of NMDA receptors at corticostriatal synapses onto CINs.

The involvement of metabotropic receptors in the potentiation of NMDA receptors (Hunt and Castillo, 2012) remains unclear at present for CINs. Interestingly, blockade rather than activation of D2 dopamine receptors appeared to enhance potentiation of the PSP amplitude. This may be ascribed to a direct D2 receptor-mediated suppression of neurotransmitter release from glutamatergic or dopaminergic terminals in the striatum (Bamford et al., 2004, 2008), or indirectly through a postsynaptic action, mobilizing endocannabinoids that in turn suppress glutamate release (Kreitzer and Malenka, 2008). Enhanced presynaptic glutamate release appears to favor the postsynaptic potentiation of NMDA receptors. But D2 receptor-mediated suppression of I*_H_* in CINs (Deng et al., 2007) may also favor the induction of LTP as described in hippocampal CA1 pyramidal neurons (Tsay et al., 2007). Therefore, the induction of potentiation of NMDA receptor transmission may depend on the interaction between post- and pre-synaptic mechanisms, while its expression may be primarily a post-synaptic effect.

What role might augmented NMDA responses play in cholinergic function in the striatum? Microdialysis studies have established that striatal acetylcholine efflux *in vivo* is increased following infusion of NMDA or AMPA and reduced by NMDA (but not AMPA) receptor blockade (Anderson et al., 1994; Consolo et al., 1996; Ikarashi et al., 1998; Knauber et al., 1999). Interestingly, we have consistently found that NMDA receptors contribute significantly to the PSP elicited by cortical afferents from the contralateral hemisphere (Oswald et al., 2009), whereas thalamic stimuli only elicit PSPs with a discernible NMDA component when delivered in bursts (Schulz et al., 2011). This suggests that cortical-driven NMDA receptor activation in CINs potently regulates striatal acetylcholine levels.

Native NMDA receptors are composed as heterotetrameric assemblies of GluN1, GluN2 (A–D) and GluN3 (A, B) subunits (Paoletti et al., 2013). CINs have been shown to express also the GluN2D NMDA receptor subunit (Bloomfield et al., 2007) that imparts NMDA receptors with a low conductance state, low sensitivity to Mg^2+^ block, and a particularly slow decay time constant (4–5 s, Cull-Candy and Leszkiewicz, 2004; Paoletti et al., 2013). Using a modulator of GluN2C/GluN2D-containing NMDA receptors (Feng et al., 2014; Zhang et al., 2014a) or an antagonist (Zhang et al., 2014b) these recent studies suggest that tonic firing of CINs is indeed increased on activation of GluN2D-containing NMDA receptors and that this modulates neurotransmitter release properties of glutamatergic, GABAergic, and dopaminergic terminals in a muscarinic receptor-mediated fashion. Interestingly, these properties were disrupted in the dopamine-depleted striatum of 6-hydroxydopamine-lesioned mice, suggesting that NMDA receptor function in CINs is significantly altered in Parkinson’s disease.

### Relevance for Reward-Related Learning

Potentiation of NMDA receptor potentials, engaged through cortical inputs, likely plays an important functional role in shaping the phasic spike pattern in CINs in response to conditioned stimuli. In behaving animals, CINs display tonic firing activity, however the presentation of salient stimuli elicits a “pause” in CIN activity that can be preceded by an initial excitation and is frequently followed by a period of rebound firing (Morris et al., 2004; Joshua et al., 2008). Recent work has highlighted the functional significance of these firing components over and above the pause, particularly in terms of encoding the mismatch between expected and actual occurrence of reward (Apicella et al., 2011; Goldberg and Reynolds, 2011).

Both cortical and thalamic pathways might contribute to these excitatory components in certain circumstances. Short and long-latency multimodal sensory inputs to the striatum from the posterior intralaminar thalamic nuclei coincide with the timing of the initial spike facilitation and the delayed rebound spike response in CINs, respectively (Matsumoto et al., 2001). However, inactivation of the intralaminar thalamus leaves intact a residual cue-induced early excitation (Matsumoto et al., 2001), which might be driven by cortical areas recently shown to report salient stimuli at very short latency (Katsuki and Constantinidis, 2012). Moreover, the reward probability signaled by the rebound excitation of CINs suggests that higher order cortical processing of expected reward value (Takahashi et al., 2011) is also made available to influence the post-pause spiking response of these interneurons (Schulz and Reynolds, 2013). The interaction of these afferent inputs from the cerebral cortex and thalamus together with those from the ventral midbrain (Brown et al., 2012; Chuhma et al., 2014; Straub et al., 2014; Wieland et al., 2014) may give rise to the variety of excitatory and inhibitory phasic firing patterns observed in CINs in different behavioral contexts (Benhamou et al., 2014).

In addition to its direct effects on CIN firing, the depolarization elicited by the NMDA receptor potential is ideally suited to evoke an AHP through deactivation of I*_H_* (Oswald et al., 2009). In the ventral striatum where glutamate co-released from dopaminergic terminals excites CINs, the firing pause in CINs with an initial burst response to pulsed activation of dopaminergic terminals had the characteristics of an AHP and was blocked on application of an NMDA receptor antagonist (Wieland et al., 2014). Importantly, we demonstrate here that plasticity of the NMDA component specifically correlates with increased AHPs. Thus, augmentation of NMDA responses rather than synaptic plasticity mechanisms *per se* could engage the AHP and induce an associated firing pause in response to a newly-conditioned stimulus. Previously, we have shown that combined HFS of purely crossed cortical PSPs and dopaminergic afferents in the substantia nigra induced an augmented slow PSP component *in vivo* (Reynolds et al., 2004) suggesting that commissural cortical synapses onto CINs show plasticity of NMDA receptor-mediated responses in the intact brain. Furthermore, the enhanced slow PSP was shown to efficiently drive a pause in the tonic discharge of CINs *in vivo* (Reynolds et al., 2004). Recruitment of AHPs in a large population of CINs through conditioning of commissural corticostriatal projections could explain the emergence of synchronized pauses throughout the striata on both sides of the brain (Aosaki et al., 1994b). Thus, pauses in spike firing in response to excitatory inputs can be driven even without the necessity for preceding spike firing (Oswald et al., 2009).

Finally, we have demonstrated here for the first time LTD occurring in commissural cortical synapses to CINs following afferent stimulation, in the absence of additional NMDA receptor activation or dopamine. This LTD was associated with a reduction in AHP amplitude, suggesting that bidirectional plasticity could not only control the emergence of synchronized pause responses but also their extinction following cue/reward dissociation.

In conclusion, we have described a novel plasticity mechanism by which cortical afferents could efficiently regulate phasic spike firing in CINs during reinforcement learning. This mechanism would explain some of the discrepancies in the firing behavior of CINs and afferent thalamic neurons whose importance for sensory responses in CINs has been widely appreciated (Matsumoto et al., 2001; Ding et al., 2010; Schulz et al., 2011).

## Author Contributions

Study concept and design: JNJR, DEO and MJO Collection of data: MJO Analysis and interpretation of data: MJO, JNJR, WK, and JMS Drafting of the manuscript: MJO and JNJR Critical revision of the manuscript: JMS, WK and DEO Study supervision: JNJR and DEO All authors approved the final version of the manuscript. All experiments except for the EPSC_NMDA_ experiments were performed in the Reynolds *in vitro* Laboratory in New Zealand. The EPSC_NMDA_ experiments were performed in the Kelsch laboratory in Germany.

## Conflict of Interest Statement

The authors declare that the research was conducted in the absence of any commercial or financial relationships that could be construed as a potential conflict of interest.

## Abbreviations

ACSF: artificial cerebrospinal fluid
AHP: afterhyperpolarization
AP5: D(-)-2-amino-5-phosphonopentanoic acid
SCH23390: R-(+)-7-chloro-8-hydroxy-3-methyl-1-phenyl-2, 3, 4, 5-tetrahydro-1H-3-benzazepine hydrochloride
CIN: cholinergic interneuron
HFS: high frequency stimulation
I*_H_*: hyperpolarization and cyclic nucleotide activated cation current
IT: intratelencephalic
LTD: long-term depression
LTP: long-term potentiation
mGluR: metabotropic glutamate receptor
NAc: nucleus accumbens
PSP: postsynaptic potential
PT: pyramidal tract
SK channel: small-conductance Ca^2+^-activated K^+^ channel
SPN: spiny projection neuron
VTA: ventral tegmental area.
